# Letter from Edinburgh

**Published:** 1885-02

**Authors:** Charles W. Purdy

**Affiliations:** Paris


### LETTER FROM EDINBURGH.

*Messrs. Editors* :—I believe I promised at Aberdeen to send you a few observations again when I reached London or the continent, and though I have not as yet remained in either of the latter sufficiently long to give you much of interest, I may yet be able to say something of interest of Scotland, where I have spent most of my time.

Of Aberdeen I have already spoken in my last letter written from there, and there is little to add. My work in the University of Aberdeen was exceedingly satisfactory to me.

Leaving Aberdeen December 26th, my next visit was to Edinburgh, where I went direct, more especially to visit the University.

One naturally expects that an institution which has given to the world such names as Grainger Stewart, Sir J. Y. Simpson, James Syme, Joseph Lister, Sir Robert Christison, and many others scarcely less widely known, would attract numbers of students from all over the world. But, I confess, I was scarcely prepared to find that the University of Edinburgh teaches as many students as all the London colleges and hospitals combined. Such, I believe, however, is the case. The University of Edinburgh, this season, has within its walls some three thousand (3,000) students, nearly two thousand (2,000) of whom are studying medicine. At first thought it would seem almost impossible to furnish teaching facilities for such a number of students in one institution. The extra-mural system of teaching is permitted in four classes out of the course, which not only relieves the classes from extra crowding, but also, through the resulting competition in lectures, tends to the attainment of a standard of greater excellence, not only among the outside teachers, but those, also, who occupy the regular chairs in the university.

Extra-mural lectures, I would say for the benefit of those not familiar with the term, means simply lectures given by an independent teacher outside, and who has no connection whatever with the university; and, as before said, a student may pursue his studies in four branches of the course with extra-mural teachers.

I met one extra-mural professor of anatomy who has three hundred and fifty students this season. One sees in the University of Edinburgh students from all over the world, of nearly every race and color, but, singular at first thought to note, nearly one-third of the whole are from England.

This latter, I am inclined to think, is due to the very high standing of the Scotch schools of medicine in the United Kingdom, or at home, in other words. It will be remembered that in Scotland there is no such thing as a half qualification; the curriculum embraces all the branches of medicine, surgery and obstetrics, while in England one goes to the college of physicians for his qualification in medicine, and to some other place for a qualification in surgery.

The new medical department of the University of Edinburgh is, no doubt, one of the finest institutions devoted to medical education in the world, and this has only been occupied now two years. Great expense and outlay has been and is still being carried on for purposes of original work. I am indebted to Professor Greenfield, Professor of Pathology, and Assistant Professor Woodhead, for the kindness of a very complete examination of their department, as well as the anatomical department of the University, on New Year's day, when, ordinarily, they are not open. The enthusiast in the "germ theory" might here revel in a veritable elysium of of microbe cultures in all their stages. It struck me, however, that in Edinburgh there was far less enthusiasm as to their etiological relations to disease than there appears to be in Germany; not that there was less interest in the subject as a pathological study, for I found quite a large room full of cultures in various stages.

The curriculum in Edinburgh is precisely the same as that at Aberdeen, of which I have already spoken in my last, and hence need not refer to it again.

It would be simply impossible for me, in the necessary limits of a letter, to give the details of the departments of this mammoth University. Its library contains 145,000 volumes, and originated in a bequest in 1580 by Mr. Clement Little, Commissary, Edinburgh, a learned citizen, and brother of the Lord Provost, who left his library to "Edinburgh and the Kirk of God." Many illustrious citizens have since bequeathed their libraries to the institution, among them the poet Drummond, of Hawthornden. In addition to the 145,000 volumes, about

2,000 volumes of MSS., many of great interest and value, and some valuable pictures and busts, are kept in the library and senate hall.

The Museum of Arts and Sciences is well worth careful examination; some of the departments are very complete, among which, especially, is that of ornithology.

Among the many pleasant memories of my visit to Edinburgh, none will be remembered longer than the great kindness and courtesy shown me by Grainger Stewart. Though his kindness to strangers is proverbial, I scarcely expected from a man, whose time is so valuable, that so much of it would be so cheerfully spent in hospitality to visitors.

The Royal Infirmary at Edinburgh, situated directly opposite the new Medical Department of the University, to the west, is a magnificent hospital, in no wise behind the great St. Thomas' at London. Essentially modern in all respects, built on the pavilion system, with spacious grounds, it contains about 600 beds, and its staff of attendants are the most careful in every detail I have yet met. It is unnecessary to say that the Royal Infirmary is not in any way *politically* conducted. The Royal Hospital for Sick Children, directly adjoining, to the east, with over a hundred beds, is also a very well conducted institution, and I was glad to find there as resident physician Dr. Anglin, a graduate of my own university, as well as fellow townsman of my native city.

I found my friend, Dr. Anglin, organizing in Edinburgh a Trans-Atlantic Club, the objects of which are:

*First.* That the members might meet at stated intervals for mutual improvement, and at all times have the opportunity of reading home papers and journals.

*Second.* To extend to new-comers, on their arrival in Edinburgh, a hearty and home-like greeting, so that they may not feel themselves strangers in a strange land, and to supply them promptly with all necessary information to aid them in their work.

I regretted that my visit to Edinburgh necessarily occurred during the mid-winter vacation, and hence I was deprived of the pleasure of hearing any of the lectures. But I may yet return for a week to Edinburgh before my return to America.

I visited, I suppose in common with most of those who go to Edinburgh, the West Port, at the west end of the Grass-market, which has acquired notoriety in connection with the series of murders committed in 1827–28 by the miscreants Burke and Hare, for the purpose of selling the bodies of their victims for dissection. I went into the cellar where these scoundrels carried on their work, and was shown over the whole place in detail.

I left Edinburgh Jan. 3d, on my way to the Continent, and stopped at Harrowgate two days with my friend Dr. Geo. Oliver. Harrowgate, it will be remembered, is the principal watering-place in the north of England. It reminds one very much of an American city, not only from its buildings, but also its roomy streets and spacious public grounds. Two hundred acres of land is reserved in the city, donated by the lord of the manor for public grounds—a privilege enjoyed by few cities in England. There are some eighty springs at Harrowgate, mostly sulphurous, and about 80,000 visitors go there each season. One spring contains chloride of iron (unusual) in a very large proportion. Dr. Oliver issues a new edition of his book on "Bedside Urinary Tests," in a few weeks, and from advance sheets which I had the pleasure of seeing, I may say that it will contain many things both new and interesting in the way of urinalysis.

My next visit was to Manchester, where, of course, I met Dr.

Roberts, and spent an agreeable evening at his house. One expects on meeting the authors of books one has read ten years or more ago, to find men of advanced years—quite old, in fact; at least such were my expectations, but in nearly every instance I have been agreeably disappointed. Dr. Roberts, for instance, and Grainger Stewart, are both men in their prime, well preserved, and not over 47, either of them, with very few gray hairs, and still doing a large amount of literary and professional work. The former (Dr. Roberts) informed me that he had just sent the last sheets of his new edition to the press the day I saw him, and Grainger Stewart presented me with a copy of his work on "Nervous Diseases," issued the same week of my visit to Edinburgh.

Again my stay in London was only transient, and I regret I am unable yet to give you anything of interest from the English medical center, and a two days' sojourn in Paris does not furnish sufficient experience to write from, hence I must reserve both for a future occasion.

CHARLES W. PURDY.

Paris, Jan. 9, 1885.

